# Integrating Assistive Technologies and Smart Home Systems in Neuromuscular Disorders: A Systematic Narrative Review of Clinical Outcomes and Occupational Therapy Perspectives

**DOI:** 10.1155/oti/3970247

**Published:** 2026-09-15

**Authors:** Zisis Lazogkas, Aggeliki Tranta, Georgia Tsakni, Daphne Bakalidou, Pinelopi Vlotinou

**Affiliations:** ^1^ Department of Occupational Therapy, University of West Attica, Athens, Greece, uniwa.gr; ^2^ Department of Physiotherapy, University of West Attica, Athens, Greece, uniwa.gr

**Keywords:** artificial intelligence, assistive technology, functional independence, neuromuscular disorders, occupational therapy, rehabilitation, smart home systems

## Abstract

**Introduction:**

Individuals living with neuromuscular disorders experience progressive functional decline that severely impacts their daily autonomy and quality of life. While restorative robotics and compensatory smart home systems (SHSs) offer potential solutions, there is a need for a synthesized clinical framework to guide their integration. This narrative review, supported by systematic search and PRISMA‐style selection, explores the synergy between these technologies and the specific role of occupational therapy in optimizing functional outcomes.

**Methods:**

A systematic narrative review was conducted by analyzing 20 primary studies focused on the application of assistive technologies in NMD populations. The synthesis categorized evidence into three primary domains: robotics and wearable systems, augmentative and alternative communication (AAC), and Internet of Things–driven smart home platforms. Clinical efficacy was evaluated based on quantitative performance metrics and qualitative user perspectives.

**Results:**

The review identifies significant functional gains associated with high‐tech interventions, including a large effect size (1.06) for robotic upper‐limb assistants. AI‐driven multimodal smart home platforms demonstrated high technical precision, with monitoring accuracies reaching approximately 94% for motor impairment stages. However, despite this high technical efficacy, real‐world utility remains complex, as clinical success is often moderated by nontechnical factors. Implementation is often hindered by barriers such as high costs, technical complexity, and privacy concerns. The findings highlight that successful integration depends on a “person–technology fit,” facilitated by occupational therapists through rigorous customization and task adaptation.

**Conclusion:**

Integrating SHSs with traditional assistive technologies significantly enhances independent living for individuals with NMD. However, to move beyond experimental success, future research must prioritize user‐centered codesign and the establishment of ethical mapping frameworks. Occupational therapists remain essential in bridging the gap between sophisticated technology and the practical, daily needs of patients.

## 1. Introduction

Neuromuscular disorders encompass a diverse range of genetic and acquired conditions that disrupt the motor unit, from the anterior horn cells to the skeletal muscles [1]. Conditions such as Duchenne muscular dystrophy, amyotrophic lateral sclerosis, and spinal muscular atrophy follow a progressive natural history characterized by muscle wasting, significant weakness, and chronic fatigue [2, 3]. These physiological impairments do not merely restrict physical movement; they impose profound activity limitations and participation restrictions that impact every facet of an individual′s life, from basic self‐care to complex community engagement [3, 4].

Individuals with NMDs frequently identify fatigue, muscle weakness, and respiratory failure as their most bothersome symptoms [2]. As degeneration advances, the transition from independent ambulation to power wheelchair dependence necessitates sophisticated technological interventions to preserve autonomy [5]. Beyond physical compensation, assistive technology serves as a critical intervention for enhancing occupational performance and facilitating engagement in meaningful life roles [6–8].

In this landscape, the occupational therapist acts as the primary bridge between clinical impairment and the maintenance of autonomy. By anchoring technology prescription in a patient′s occupational profile—targeting specific issues like the inability to operate home controls independently—therapists ensure that AT is not just a hardware solution but a means of restoring participation in chosen life roles [9, 10]. This bridge is essential across the AT continuum, which spans from low‐tech specialized utensils to high‐tech powered exoskeletons and speech‐generating interfaces [11–13]. Simultaneously, smart home systems utilize the Internet of Things and ambient assisted living frameworks to create adaptable environments [14]. These systems integrate sensors and voice‐activated hubs to provide hands‐free control over household functions, thereby fostering independence and reducing the caregiver burden associated with recurrent requests for help [15, 16].

### 1.1. Research Gap

Despite the documented potential of these technologies, the literature lacks a clear framework for managing long‐term integration in progressive conditions. Three specific gaps limit current clinical utility:

1. *Longitudinal adherence gap*: Most studies focus on short‐term functional improvements (typically 4–8 weeks) rather than tracking how technology use evolves across the full NMD disease trajectory.
2. *Transition management gap*: There is a significant lack of evidence‐based guidance for the transition from restorative robotics to compensatory smart home systems. The literature treats these interventions in isolation rather than as a continuous clinical pathway.
3. *The usability-equipment burden gap*: A persistent pattern of device abandonment occurs when users feel the “equipment burden”—the cognitive and physical effort of operating the system—outweighs the functional gain.

## 2. Methods

### 2.1. Search Strategy and Database Selection

This study uses a narrative‐review methodology supported by a systematic search procedure, conducted across four databases, to examine the current clinical and functional status of assistive‐technology interventions for individuals with neuromuscular disorders. A comprehensive and systematic literature search was executed across four primary academic databases—PubMed, Scopus, Web of Science, and Google Scholar—with the final query successfully completed on March 5, 2026. The search architecture utilized combinations of Boolean operators and truncated keywords structured across three core methodological clusters: population (neuromuscular disorders, amyotrophic lateral sclerosis, and Duchenne muscular dystrophy), technology (assistive technology, smart home systems, and robotics), and clinical outcomes (occupational therapy and functional independence).

The review consideration framework encompassed meta‐analyses, randomized controlled trials (RCTs), nonrandomized designs, and descriptive case reports published within a specific multiyear window from 2018 to 2026. Literature selection followed a rigorous dual‐reviewer screening protocol. Two reviewers independently evaluated all retrieved titles and abstracts against the predetermined eligibility criteria, followed by a collaborative full‐text evaluation of the shortlisted papers. Any screening or inclusion disagreements were resolved systematically through targeted discussion and consensus. The complete multistage study selection and attrition process is fully documented via a standard PRISMA flow diagram. Finally, methodological quality was evaluated using a split strategy: Primary empirical studies were formally appraised using the Mixed Methods Appraisal Tool (MMAT) v2018, whereas secondary review‐level evidence was critically evaluated based on its respective methodological design and appropriate reporting components.

### 2.2. Inclusion and Exclusion Criteria

Study selection was based on specific criteria to ensure the clinical relevance of the data. We included peer‐reviewed original research, systematic or scoping reviews, and detailed clinical case reports that focused on populations with neuromuscular disorders. Additionally, eligible studies had to report on functional, usability, or ethical outcomes and be published in the English language.

On the other hand, we excluded conference abstracts and engineering‐only papers that did not include human clinical trials. We also excluded studies focusing on general aging or cognitive disabilities, such as Alzheimer′s disease, unless they had direct relevance to motor disabilities. Finally, non‐peer‐reviewed sources were omitted from this review.

### 2.3. Quality Appraisal Strategy

To evaluate the methodological quality of the 20 included studies, we implemented a targeted appraisal strategy based on study design. Primary empirical studies (*n* = 12) were formally appraised using the MMAT, Version 2018. Studies were categorized into specific design tracks: qualitative, quantitative randomized, quantitative nonrandomized, and quantitative descriptive. Conversely, because the MMAT is strictly designed for primary empirical data, the remaining eight review‐level articles were classified as not applicable (N/A) for formal quality scoring. To maintain evidence transparency, these review‐level papers were evaluated based on their respective methodological designs, with systematic reviews and meta‐analyses specifically screened for compliance with standard reporting components.

### 2.4. PRISMA Flow Description

This study is a narrative review supported by a systematic search procedure, with the literature selection process modeled on the PRISMA framework. A total of 2115 records were identified through database searching. After the removal of 273 duplicate records, 1842 studies remained for title and abstract screening. During this phase, 1620 records were excluded as they did not meet the inclusion criteria. Subsequently, 222 full‐text articles were assessed for eligibility. Of these, 202 studies were excluded for several reasons, including lack of focus on neuromuscular disorders (*n* = 86), engineering‐based studies without clinical participants (*n* = 57), conference abstracts or studies with insufficient methodological detail (*n* = 34), and non‐peer‐reviewed sources (*n* = 25). Twenty studies met the inclusion criteria and were included in the final synthesis. Specifically, among the 20 included papers, the final synthesis incorporates one pooled meta‐analysis, one multicentric RCT, 10 nonrandomized/quantitative descriptive studies, two qualitative investigations, and six systematic/scoping reviews.

### 2.5. Clinical Assessment Tools and Permissions

This review evaluates and recommends the use of specific validated instruments for clinical practice in NMD. Specifically, the Canadian Occupational Performance Measure (COPM) is discussed as a primary tool for client‐centered goal setting and outcome measurement [9]. The System Usability Scale (SUS) is utilized within the synthesized evidence to quantify the technical acceptability of assistive devices [17]. In accordance with journal guidelines, the authors acknowledge that these tools are used as established clinical instruments. The COPM is a proprietary measure, and its use in the context of this research and recommended clinical applications adheres to the licensing and training requirements set forth by the authors and official distributors [9]. Similarly, the SUS is a standard industry tool used here for descriptive clinical analysis [17]. No modifications were made to the original scales, and all tool‐related data extracted from the primary studies were used with respect to the original authors′ permissions and ethical approvals.

## 3. Assistive Technologies in NMD: A Scientific Synthesis

### 3.1. Robotics and Wearable Systems

Clinical utility in robotics is divided by the anatomical focus of the intervention. High‐validity meta‐analyses indicate that upper‐limb robotic assistants provide substantial functional benefits, yielding a large effect size of 1.06. These results, however, do not translate uniformly across the NMD spectrum. Lower‐limb exoskeletons remain supported only by short‐term, small‐scale trials that lack the longitudinal depth found in upper‐extremity research [18, 19].

A notable selection bias limits the generalizability of these findings; participants in these trials typically possess residual motor function, excluding those in advanced disease stages where the motor unit disruption described by Zambon et al. is most severe. This oversight ignores the physiological reality of progressive muscle wasting, which eventually renders restorative robotics obsolete. For these individuals, the usability gap widens as the physical demands of operating a wearable system conflict with the need for energy conservation.

### 3.2. Augmentative and Alternative Communication (AAC): The Precision Pattern

The landscape of AAC is undergoing a transition from hardware‐dependent tools, such as oculography and physical switches, toward contactless multimodal interfaces. Technical performance in this domain follows a “precision pattern,” with modern AI‐driven models achieving monitoring and interaction accuracies as high as 94.98% [20, 21].

Technical accuracy does not ensure clinical durability. A profound discrepancy exists between these high‐precision metrics and the frequent device abandonment observed in late‐stage conditions. The primary driver of this failure is the equipment burden imposed by sophisticated interfaces. When users experience the chronic fatigue and muscle weakness characteristic of NMDs [2, 3], the effort required to sustain interaction with complex AAC systems often exceeds the perceived functional gain. While Grajo and Boisselle frame AT as a critical intervention for maintaining participation, these high‐precision tools only succeed if they account for the user′s diminishing physical and cognitive reserves.

### 3.3. Quality Appraisal and Risk of Bias

The methodological quality of the evidence varies significantly across the analyzed technology categories. Robotics research demonstrates high internal validity through RCTs, yet it frequently suffers from selection bias because most clinical participants retain residual motor function [19, 22]. In contrast, smart home systems and AAC research are dominated by descriptive studies and case reports. This introduces a notable reporting bias, where immediate technical successes are favored over long‐term data regarding device abandonment [23–25] (Table 1).

**Table 1:** Clinically annotated synthesis and methodological categorization (*N* = 20).

Author	Country	Study design	OT framework/model	Population (*N*)	Key clinical metrics and results
Atashzar et al. [26]	United States/Canada	Systematic review	Intelligent robotics	Not applicable (theoretical)	Intelligent robots facilitate remote assistance but require human oversight.
Ding et al. [27]	United States	Qualitative	Professional perspective	*N* = 16 professionals	Professionals view mainstream smart technology as a viable, low‐cost alternative to specialized medical AT.
Felber et al. [28]	Switzerland	Systematic review	Ethical mapping	*N* = 156 papers	Mapped ethical tensions in autonomy, privacy, and responsibility in smart home healthcare.
Gandolla et al. [22]	Italy	Meta‐analysis	ICF framework	*N* = 345 participants	Effect size: 1.06 for upper‐limb functional tasks in people with neuromuscular diseases.
Giansanti [13]	Italy	Narrative review	PEO (person–environment–occupation)/occupational performance	*N* = 24 papers	Synthesis confirmed that AT enhances mobility and quality of life across care settings.
Knippenberg et al. [17]	Belgium	Mixed methods	i‐ACT/client‐centered	*N* = 17 participants	SUS score ≥ 73.8/100; high intrinsic motivation (≥ 5.2/7.0) for training.
Linse et al. [23]	Germany	Quantitative descriptive	Resilience and social participation	*N* = 101 participants	Late‐stage introduction of high‐tech AAC significantly increases device abandonment risks.
Longatelli et al. [19]	Italy	Multicentric RCT	ICF framework	*N* = 36 participants	Passive and semiactive supports improved functional gain at *p* < 0.01.
Martínez et al. [29]	Spain	Quantitative non‐RCT	Psychosocial	*N* = 73 participants	Teleassistance significantly improved quality of life and social participation in rare NMD.
Morita et al. [30]	Canada	Scoping review	Interdisciplinary	*N* = 49 papers	Lack of interdisciplinary collaboration; technology development currently dominates human‐centric design.
Nishida et al. [24]	Japan	Case report	Social participation	*N* = 1 participant	Noncontact gesture switches enhanced communication speed and social agency in advanced DMD.
Pousada et al. [10]	Spain	Cross‐sectional	Client‐centered	*N* = 24 participants	Mean FIM motor: 61.7/91; 87.5% of users faced architectural barriers in their homes.
Rodrigues et al. [8]	Brazil	Theoretical	Activity and participation	Not applicable (theoretical)	OT intervention increases success rates of matching people with technology through clinical reasoning.
Rodriguez‐Fernández et al. [18]	Spain	Systematic review	Gait training	*N* = 14 papers	Summarized evidence for wearable exoskeletons in enhancing gait and social participation.
Sanzo′ et al. [31]	Italy	Quant. descriptive	Resilience and social participation	*N* = 59 participants	28.81% depression rate; high resilience scores negatively correlated with psychopathology.
Tang et al. [20]	China	Technical pilot	Pattern recognition	*N* = 10 participants	AI‐driven platforms reached up to 94% accuracy in classifying motor recovery stages.
Tavares et al. [25]	Portugal	Case study	PEO/occupational performance	*N* = 1 (caregiver)	Smart speakers as ECUs effectively reduced caregiver burden in severe motor dependence.
Wang et al. [32, 33]	China	Systematic review	PRISMA/independence	*N* = 27 papers	Confirmed that SHS alleviates caregiver burden and enhances functional independence.
Xu et al. [21]	United States	Technical pilot	Noncontact monitoring	*N* = 62 participants	Soli radar achieved an MAE of 1.69 bpm for contactless sleep tracking.
Yaffe et al. [34]	Australia	Qualitative	Appreciative inquiry	*N* = 9 participants	“Person–technology fit” and “choice/control” are primary indicators of freedom for home AT users.

Regarding the formal MMAT evaluation, all 12 primary empirical studies successfully met both preliminary core screening criteria by establishing a clearly defined research question and utilizing data collection methods directly suited to their objectives. This eligibility allowed all 12 papers to qualify for full thematic quality scoring across their respective design categories, yielding quality scores between 80% and 100%. Qualitative evaluations and quantitative randomized trials achieved full scores due to robust protocols and high data coherence [19, 27, 34]. Meanwhile, lower scores in certain quantitative descriptive designs were primarily driven by small, non‐representative sample sizes, which is an expected limitation in rare neuromuscular disorder research [23–25]. The item‐by‐item scoring breakdown is detailed in Supporting Information 1: Table S1.

## 4. Smart Home Integration Models

### 4.1. Smart Home Systems: Immediate Autonomy Versus Evidence Level

Smart home integration for NMD follows two distinct trajectories based on the technological medium: ambient Internet of Things sensors and active robotic assistants. IoT architectures, as analyzed by Ding et al. and Tavares et al., prioritize immediate environmental autonomy through low‐friction modifications. These deployments often leverage larger cohorts than robotics trials, yet they frequently lack the longitudinal depth required to track declining physical function over time. Conversely, robotic‐centered homes [28, 35] focus on physical task execution through high‐rigor, small‐sample designs (typically *n* < 10). While these robotic systems demonstrate high technical accuracy, they suffer from immature outcome measures that fail to capture real‐world durability outside of controlled environments. The current evidence base suggests that while robotics offers task‐specific support, it fails to match the environmental–control scalability of IoT, leaving the optimal timing for home‐based robotic integration an unresolved clinical question for progressive conditions.

### 4.2. Pattern Comparison: IoT Versus Robotics

This technological split mirrors a fundamental tension between restorative and compensatory occupational therapy frameworks. Robotic integration typically adopts an ICF‐based restorative perspective, attempting to bridge the motor gap through mechanical assistance [26, 30]. This approach eventually conflicts with the physiological reality of NMD progression, where severe muscle wasting renders active assistance less viable [1, 2]. In contrast, IoT systems operate within a PEO‐based compensatory framework, shifting the burden from the impaired body to the modified environment [7, 9]. This shift is critical as patients transition from physical‐function metrics toward environmental‐autonomy metrics in later disease stages. Despite these theoretical differences, the field lacks a validated framework to guide therapists in managing the transition from restoration‐heavy robotics to compensation‐heavy IoT as the user′s physical reserves diminish (Table 2).

**Table 2:** Comparison of clinical patterns by technology type. The following table provides a side‐by‐side comparison of how these two patterns are evaluated and implemented within occupational therapy practice.

Feature	Restoration pattern	Compensation pattern (IoT/SHS)
Primary goal	Enhancing motor capability and range of motion ([19]; [22])	Facilitating task completion through environmental control ([27]; [25])
Clinical focus	Body‐level function and physical participation ([19]; [18])	Occupational performance and aging‐in‐place ([34]; [32, 33])
Example tech	Robotic arms, wearable exoskeletons, and haptics ([22]; [26])	Smart speakers, automated lighting, and climate control ([27]; [25])
OT metric	FIM motor scores and joint ROM ([19]; [10])	COPM performance/satisfaction and caregiver burden ([25]; [9])
Major advantage	Promotes neuroplasticity; functional gain effect size: 1.06 ([26]; [22])	Low cost, high flexibility, and immediate user autonomy ([34]; [27])
Major barrier	High cost and technical setup complexity [22]	Privacy concerns and internet dependency ([30]; [28])

A cross‐cutting issue in the literature is the heterogeneity of NMD progression. Most studies fail to provide a stratified analysis based on the stage of the disease. Furthermore, the follow‐up duration across all categories is insufficient to capture the “shifting needs” of patients as their motor status declines, suggesting a need for longitudinal real‐world data rather than short‐term lab‐based metrics [28, 36].

## 5. Clinical Outcomes and Functional Impact

A critical synthesis of current evidence reveals a split between technical accuracy and real‐world clinical utility. In robotics, while meta‐analyses report a substantial pooled effect size of 1.06 for upper‐limb ADL performance [22], these findings suffer from a significant selection bias. Most participants in successful robotics trials possess residual motor function, which excludes individuals in advanced disease stages where muscle wasting is most severe [1]. This creates a “restorative ceiling” where robotic interventions lose efficacy as the physiological capacity for active assistance diminishes.

Similarly, in communication and environmental control, AI‐driven multimodal platforms achieve technical accuracies as high as 94% [20]. However, this high precision does not always correlate with longitudinal satisfaction. While psychosocial interventions and teleassistance improve quality of life and reduce social isolation [29], the clinical trajectory often shifts from high‐precision interaction to low‐friction compensation. The evidence suggests that for NMD populations, the durability of an intervention depends less on its technical sophistication and more on its ability to adapt as motor units fail. Future outcomes must therefore move beyond purely physical metrics to include client‐perceived performance and satisfaction [9].

## 6. Role of Occupational Therapy

The occupational therapist′s role has evolved from simple device administration to a critical management of the “person–technology fit.” This fit is not static; it requires continuous clinical auditing of the user′s remaining motor functions and the environment in which they operate. Utilizing tools like the COPM allows therapists to quantify functional gains based on the user′s personal priorities rather than purely technical benchmarks [9, 17].

A critical component of this role is the environmental audit. Evidence shows that 87.5% of NMD users live in homes with significant architectural barriers that compromise the placement and functionality of AT [10]. The therapist must synthesize these physical constraints with the user′s motivational and clinical indices. Systems with a SUS score above 73.8 are significantly more likely to be integrated into daily routines [17]. By addressing environmental factors alongside hardware customization, the OT ensures that smart home systems are successfully adopted rather than abandoned, bridging the gap between a generic technical solution and a tailored clinical intervention [35, 36].

## 7. Barriers and Ethical Considerations

### 7.1. Synthesis of Implementation Barriers

The integration of assistive technologies in NMD is frequently hindered by nontechnical factors that can lead to high rates of device abandonment if not addressed by the clinical team. For practitioners, the primary takeaway is the shift from viewing these hurdles as technical “bugs” to treating them as clinical “occupational barriers” that require specific therapeutic interventions (Table 3).

**Table 3:** Implementation barriers and actionable OT strategies. This table serves as a primary resource for practitioners, mapping common barriers to evidence‐based clinical solutions. AT implementation barrier and mitigation matrix.

Implementation barrier	Clinical evidence and detail	Actionable OT mitigation strategy
Architectural incompatibility	Barriers detected in 87.5% of homes, preventing physical AT placement [10].	Conduct a structured home accessibility audit prior to device selection to ensure the environment supports the hardware footprint ([36]; [10]).
System unreliability	Frequent frustration with voice‐recognition failures and internet instability [34].	Develop a “fail‐safe protocol” including analogue backups and secondary triggers to ensure safety during connectivity outages ([34]; [15]).
Psychosocial burden	High prevalence of depression (28.81%) and anxiety linked to perceived loss of control [31].	Apply the COPM for goal alignment to ensure technology addresses high‐priority occupations, increasing resilience and adherence ([31]; [37]).
Input and cognitive fatigue	Eye‐tracking precision often leads to ocular and cognitive exhaustion [16].	Implement hybrid input methods, such as noncontact gesture switches for basic tasks, to reduce eye strain ([24]; [16]).
Ethical concerns	Tension between the desire for autonomy and the discomfort of constant monitoring [28].	Facilitate “meaningful transparency” through human‐centered AI education, ensuring users understand monitoring parameters ([38]; [28]).

### 7.2. Ethical Mapping

The integration of smart home systems creates a constant tension between patient safety and personal privacy. Although continuous, noncontact monitoring for metrics like heart rate and fall detection provides life‐saving data, it also introduces the risk of constant surveillance. This continuous monitoring can inadvertently turn a home into a clinical environment rather than a private space [28, 32, 33]. Occupational therapists must navigate these issues by advocating for human‐centered design and transparent systems. This ensures that patients retain choice and control over their environment, which users identify as the primary indicator of freedom when using electronic assistive technology at home [34, 38].

To achieve ethical integration, practitioners must establish clear clinical guidelines that go beyond simple equipment installation. First, therapists need to ensure that patients provide informed consent specifically for uploading data to clinical platforms, such as clinical patient management systems, in full compliance with General Data Protection Regulation (GDPR) standards [39]. Second, data governance protocols must be maintained through secure methods like two‐factor authentication, alongside a clear separation between identifiable data used for direct care and anonymized data used for research purposes [40]. Finally, clinicians must carefully balance informational privacy, which concerns data sharing, with physical privacy, which involves monitoring actual activities within the home, to prevent the unnecessary medicalization of the patient′s private living environment [41, 42].

## 8. Future Research Directions

### 8.1. Methodological Shifts

Future research must prioritize longitudinal studies that track the ecological validity of smart home systems over the progressive course of neuromuscular disorders. Current evidence is often limited to cross‐sectional “snapshots” of technology use, which fails to account for the changing physical and cognitive needs of the user [35, 36]. There is a critical need for standardized reporting of technical reliability and user‐perceived satisfaction using validated tools like the COPM to build a robust evidence base that can inform clinical guidelines [9, 17].

### 8.2. The Necessity of Codesign: From Implementation to Innovation

A significant shift is required in the development lifecycle of assistive technologies, moving from a “technology‐push” model to a “human‐centered pull” model. Co‐design research must transition the occupational therapist from an “end‐stage implementer”—who simply adapts existing products—to an “upstream designer” involved in the initial ideation and prototyping phases [38].

Evidence suggests that when OTs are involved in the development of AI‐driven and smart home systems, the resulting technologies are more ethically aligned and better integrated into the user′s occupational identity [38, 42]. By participating in the proactive analysis of ethical concerns and functional requirements during the design stage, OTs can ensure that developers prioritize “choice and control” as central technical requirements rather than secondary features [34, 42]. This collaborative approach ensures that the technology is “occupation‐ready” upon market release, reducing the high rates of device abandonment currently seen in complex disability populations [10, 35].

### 8.3. Limitations

A critical appraisal of the current NMD literature reveals several methodological limitations that restrict the real‐world application of assistive technologies. First, a pronounced selection bias skews current data; study participants often possess baseline high technological literacy and stable social support structures, which do not reflect the broader, more isolated NMD population [43]. Second, sample sizes in quantitative trials remain small—frequently under 40 participants—which restricts the generalizability of reported functional gains [19]. Third, a significant longitudinal evidence gap persists. Most efficacy data are derived from short‐term intervention windows, often limited to 6 weeks [17], completely failing to monitor device abandonment rates as physical weakness inevitably progresses over time [23]. Furthermore, current research is heavily siloed within technical disciplines, where engineering metrics of system performance frequently overshadow human‐centric, interdisciplinary design principles [30]. Technical vulnerabilities, such as internet dependency in smart home systems and voice‐recognition failures, introduce safety risks during connectivity outages that remain poorly documented in clinical frameworks [34].

To overcome these barriers, future research must shift from a technology‐push model to a client‐centered, needs‐led design process. Methodologically, researchers must prioritize longitudinal designs exceeding 12 months to systematically track technology adherence across varying stages of disease progression [23, 35]. Crucially, the development lifecycle of these technologies must transition toward participatory codesign. Rather than positioning the occupational therapist as an end‐stage implementer who merely adapts pre‐existing devices, clinical insights must be integrated upstream during the initial ideation and algorithmic prototyping phases [38]. Proactive codesign ensures that “choice, control,” and ethical data privacy are treated as core technical requirements rather than secondary features, drastically mitigating the risk of early device abandonment [10, 34, 42].

## 9. Conclusions and Clinical Implications

Integrating smart home systems and assistive technology transforms a patient′s living space from a passive environment into an active, supportive home environment that preserves autonomy. However, clinical utility is not determined by the sophistication of the hardware but by the precision of the person–technology fit. This review emphasizes that technology provision must follow a staged, clinical progression aligned with the user′s diminishing physical and cognitive reserves. Early‐stage interventions should leverage low‐effort ambient controls for energy conservation, mid‐stage plateaus may integrate active robotics, and advanced stages require highly precise, contactless alternative communication interfaces introduced proactively before speech loss occurs.

By utilizing instruments like the COPM, occupational therapists provide the necessary clinical link between technical features and meaningful daily goals, ensuring that the equipment burden does not eclipse the user′s functional independence. Finally, successfully moving these technologies from experimental setups to sustainable home settings requires a balanced approach that mitigates architectural barriers, reduces caregiver stress, and navigates the ethical tension between continuous safety monitoring and personal privacy.

**Nomenclature**
AAC: augmentative and alternative communication
AAL: ambient assisted living
ADL: activities of daily living
AI: artificial intelligence
ALS: amyotrophic lateral sclerosis
AT: assistive technology
BCI: brain–computer interface
COPM: Canadian Occupational Performance Measure
DMD: Duchenne muscular dystrophy
ECU: environmental control unit
IoT: Internet of Things
NMD: neuromuscular disorder
OT: occupational therapy/occupational therapist
QoL: quality of life
ROM: range of motion
SHS: smart home system
SMA: spinal muscular atrophy
SUS: System Usability Scale

## Author Contributions

Z.L.: conceptualization, methodology, software, formal analysis, investigation, data curation, writing—original draft, visualization, and project administration. A.T.: investigation and writing—review and editing. G.T.: investigation and writing—review and editing. D.B.: writing—review and editing. P.V.: supervision and writing—review and editing.

## Funding

This research was supported/funded by the Special Account for Research Grants (ELKE) of the University of West Attica.

## Conflicts of Interest

The authors declare no conflicts of interest.

## Supporting Information

Additional supporting information can be found online in the Supporting Information section.

## Supporting information


**Supporting Information 1** Table S1: Methodological quality appraisal matrix (MMAT 2018).

**Supporting Information 2**  

## Data Availability

The data that support the findings of this study are available in the supporting information of this article.

## Appendix A Detailed Search Strategy and Boolean Architecture

To guarantee full methodological transparency and reproducibility, the exact operational syntax, Boolean logic, and truncation parameters utilized during the database query process are detailed below. The literature search was constructed around three distinct thematic clusters: target neuromuscular pathologies, specific technological interventions, and relevant clinical or occupational therapy outcomes.

Search Cluster Breakdown:

• Cluster 1 (Target Pathology): Focuses on neuromuscular conditions and includes terms such as neuromuscular disorders (MeSH), muscular dystrophy, amyotrophic lateral sclerosis (ALS), Duchenne, and spinal muscular atrophy (SMA).
• Cluster 2 (Intervention Technology): Encompasses assistive devices and smart environments, including terms such as assistive technology (MeSH), smart home systems, artificial intelligence (MeSH), ambient assisted living, robotic exoskeletons, eye tracking, and the Internet of Things (IoT).
• Cluster 3 (Clinical/Occupational Outcome): Target goals and frameworks, including occupational therapy (MeSH), rehabilitation, functional independence, and quality of life.

The exact syntax executed within each database query engine to intersect these clusters is presented in Table A1.

**Table A1:** Database‐Specific Search Strings and Structural Syntax.

Database	Specific Search String
PubMed	("assistive technology"[MeSH Terms] OR "smart home"[All Fields] OR "artificial intelligence"[MeSH Terms]) AND ("neuromuscular disorders"[MeSH Terms] OR "muscular dystrophy"[All Fields] OR "amyotrophic lateral sclerosis"[All Fields] OR "spinal muscular atrophy"[All Fields]) AND ("occupational therapy"[MeSH Terms] OR "rehabilitation"[All Fields])
Scopus	TITLE‐ABS‐KEY(("assistive technology" OR "smart home" OR "ambient assisted living" OR "IoT") AND ("neuromuscular disorder∗" OR "Duchenne" OR "ALS" OR "SMA") AND ("occupational therapy" OR "functional independence"))
Web of Science	TS=(("assistive technology" OR "robotic exoskeleton" OR "eye tracking") AND ("neuromuscular disease") AND ("occupational therapy" OR "quality of life"))
Google Scholar	("assistive technology" OR "smart home systems") AND ("neuromuscular disorders") AND ("occupational therapy implications") custom range filter (2018–2026)
