# Supplementary material for: Integrating Assistive Technologies and Smart Home Systems in Neuromuscular Disorders: A Systematic Narrative Review of Clinical Outcomes and Occupational Therapy Perspectives
Source: Occup Ther Int. 2026 Sep 15;2026:3970247. doi: 10.1155/oti/3970247 (PMC13576904; doi:10.1155/oti/3970247)
Supplement: Supplementary file 1 [file OTI-2026-3970247-s002.docx]

**Identification of studies via databases and registers**

Records removed *before screening*:

Duplicate records removed (n = 273)

Records marked as ineligible by automation tools (n = 0)

Records removed for other reasons (n = 0)

Records identified from*:

Databases (n = 2,115)

Registers (n = 0)

**Identification**

Records screened

(n = 1842)

Records excluded**

(n = 1,620)

Reports sought for retrieval

(n = 222)

Reports not retrieved

(n =0 )

**Screening**

Reports excluded:

Not focused on neuromuscular disorders (general ageing populations or cognitive conditions such as Alzheimer’s disease) **n = 86**

Engineering or technical studies without human participants or clinical evaluation **n = 57**

Conference abstracts or studies with insufficient methodological detail **n = 34**

Non–peer-reviewed sources (editorials, opinion papers, non-indexed reports) **n = 25**

**Total:** 202

Reports assessed for eligibility

(n = 222)

Studies included in review

(n =20 )

**Included**
