# Supplementary material for: Integrating Assistive Technologies and Smart Home Systems in Neuromuscular Disorders: A Systematic Narrative Review of Clinical Outcomes and Occupational Therapy Perspectives
Source: Occup Ther Int. 2026 Sep 15;2026:3970247. doi: 10.1155/oti/3970247 (PMC13576904; doi:10.1155/oti/3970247)
Supplement: Supplementary file 2 [file OTI-2026-3970247-s001.zip › Supplementary Table 1 Methodological Quality Appraisal.1/Search strings.docx]

| Database | Specific Search String |
| --- | --- |
| **PubMed** | ("assistive technology"[MeSH Terms] OR "smart home"[All Fields] OR "artificial intelligence"[MeSH Terms]) AND ("neuromuscular disorders"[MeSH Terms] OR "muscular dystrophy"[All Fields] OR "amyotrophic lateral sclerosis"[All Fields] OR "spinal muscular atrophy"[All Fields]) AND ("occupational therapy"[MeSH Terms] OR "rehabilitation"[All Fields]) |
| **Scopus** | TITLE-ABS-KEY(("assistive technology" OR "smart home" OR "ambient assisted living" OR "IoT") AND ("neuromuscular disorder*" OR "Duchenne" OR "ALS" OR "SMA") AND ("occupational therapy" OR "functional independence")) |
| **Web of Science** | TS=(("assistive technology" OR "robotic exoskeleton" OR "eye tracking") AND ("neuromuscular disease") AND ("occupational therapy" OR "quality of life")) |
| **Google Scholar** | ("assistive technology" OR "smart home systems") AND ("neuromuscular disorders") AND ("occupational therapy implications") |
