# Supplementary material for: Integrating Assistive Technologies and Smart Home Systems in Neuromuscular Disorders: A Systematic Narrative Review of Clinical Outcomes and Occupational Therapy Perspectives
Source: Occup Ther Int. 2026 Sep 15;2026:3970247. doi: 10.1155/oti/3970247 (PMC13576904; doi:10.1155/oti/3970247)
Supplement: Supplementary file 2 [file OTI-2026-3970247-s001.zip › Supplementary Table 1 Methodological Quality Appraisal.1/Supplementary Table 1 Methodological Quality Appraisal.1.docx]

**Supplementary Material**

**Supplementary Table 1: Methodological Quality Appraisal Matrix (MMAT 2018)**

| Author | Design Category | S1 | S2 | Q1 | Q2 | Q3 | Q4 | Q5 | Quality (%) |
| --- | --- | --- | --- | --- | --- | --- | --- | --- | --- |
| Atashzar et al., 2021 | Systematic Review | - | - | - | - | - | - | - | N/A |
| Ding et al., 2021 | Qualitative | Y | Y | Y | Y | Y | Y | Y | 100% |
| Felber et al., 2023 | Systematic Review | - | - | - | - | - | - | - | N/A |
| Gandolla et al., 2020 | Meta-analysis | - | - | - | - | - | - | - | N/A |
| Giansanti, 2025 | Narrative Review | - | - | - | - | - | - | - | N/A |
| Knippenberg et al., 2021 | Mixed Methods | Y | Y | Y | Y | Y | Y | C | 90% |
| Linse et al., 2018 | Quant. Descriptive | Y | Y | Y | Y | Y | N | Y | 80% |
| Longatelli et al., 2021 | Quant. Randomized | Y | Y | Y | Y | Y | Y | Y | 100% |
| Martínez et al., 2021 | Quant. Non-Randomized | Y | Y | Y | Y | Y | Y | N | 80% |
| Morita et al., 2023 | Scoping Review | - | - | - | - | - | - | - | N/A |
| Nishida et al., 2025 | Quant. Descriptive | Y | Y | Y | Y | Y | N | Y | 80% |
| Pousada et al., 2019 | Quant. Descriptive | Y | Y | Y | Y | Y | Y | Y | 100% |
| Rodrigues et al., 2023 | Theoretical | - | - | - | - | - | - | - | N/A |
| Fernández et al., 2021 | Systematic Review | - | - | - | - | - | - | - | N/A |
| Sanzo’ et al., 2024 | Quant. Descriptive | Y | Y | Y | Y | Y | Y | Y | 100% |
| Tang et al., 2024 | Quant. Descriptive | Y | Y | Y | Y | Y | Y | Y | 100% |
| Tavares et al., 2024 | Quant. Descriptive | Y | Y | Y | Y | Y | N | Y | 80% |
| Wang et al., 2024 | Systematic Review | - | - | - | - | - | - | - | N/A |
| Xu et al., 2023 | Quant. Descriptive | Y | Y | Y | Y | Y | Y | Y | 100% |
| Yaffe et al., 2025 | Qualitative | Y | Y | Y | Y | Y | Y | Y | 100% |

**Notes & Abbreviations:** Y = Yes (Criterion met); N = No (Criterion not met); C = Cannot tell; N/A = Not Applicable (Review-level studies omitted from primary empirical appraisal).

- **Screening Criteria:** S1 = Are there clear research questions?; S2 = Do the collected data allow to address the research questions?
- **Core Quality Criteria (Q1–Q5):** Appraised dynamically based on the specific methodological requirements of each MMAT 2018 design category (Qualitative, Quantitative Randomized, Quantitative Non-Randomized, and Quantitative Descriptive).

**Methodological Quality and Appraisal Rationale**

The methodological appraisal reveals specific quality trends across the different study designs. The included qualitative investigations demonstrated high methodological rigor. Specifically, the studies by Ding et al. (2021) and Yaffe et al. (2025) achieved a score of 100% because they clearly established the clinical and personal context, utilized appropriate qualitative data collection methods—such as semi-structured interviews and World Cafés—and maintained full coherence between the field data and final interpretation. For instance, Yaffe et al. (2025) effectively employed an Appreciative Inquiry framework to investigate the "Person-Technology Fit" as a primary indicator of user independence. Similarly, the quantitative randomized controlled trial by Longatelli et al. (2021) fulfilled all MMAT criteria (100% score), utilizing a robust multicentric randomization protocol and maintaining high adherence to outcome assessments, which provided strong internal validity for the reported functional gains.

For the mixed-methods literature, Knippenberg et al. (2021) received a quality score of 90% due to an unclear designation on the final criterion (C on Q5). While the study provided a strong methodological rationale for integration by combining quantitative usability scores with qualitative motivation indices, it did not fully address or explore the technical divergences between qualitative motivation and quantitative usability outcomes. Such integration issues represent a recognized challenge within mixed-methods rehabilitation research.

Regarding the quantitative descriptive and non-randomized studies, variations in scoring were primarily linked to sampling parameters. Studies by Pousada et al. (2019), Sanzo’ et al. (2024), Tang et al. (2024), and Xu et al. (2023) met all quality benchmarks. These trials effectively utilized validated clinical measurement tools, such as the Functional Independence Measure motor score or standardized resilience scales, to evaluate user outcomes. Conversely, the descriptive and non-randomized studies by Linse et al. (2018), Martínez et al. (2021), Nishida et al. (2025), and Tavares et al. (2024) scored 80%. This reduction was driven by a negative appraisal on sample representativeness (N on Q4). These studies relied on restricted or small sample sizes, which inherently limits the generalizability of the findings to the broader target population; however, this remains a standard and expected constraint for clinical evaluations involving rare neuromuscular disorders.

Finally, the remaining eight review-level articles—encompassing systematic reviews, scoping reviews, narrative overviews, and meta-analyses—were excluded from formal MMAT scoring since the tool is restricted to primary empirical data. To maintain reporting integrity, these papers were instead cross-checked for PRISMA reporting guidelines compliance, verifying that they maintained transparent search strings, defined eligibility criteria, and dual-reviewer screening protocols to ensure evidence transparency.

**Appendix A: Detailed Search Strategy and Boolean Architecture**

To guarantee full methodological transparency and reproducibility, the exact operational syntax, Boolean logic, and truncation parameters utilized during the database query process are detailed below. The literature search was constructed around three distinct thematic clusters: target neuromuscular pathologies, specific technological interventions, and relevant clinical or occupational therapy outcomes.

Search Cluster Breakdown:

- Cluster 1 (Target Pathology): Focuses on neuromuscular conditions and includes terms such as neuromuscular disorders (MeSH), muscular dystrophy, amyotrophic lateral sclerosis (ALS), Duchenne, and spinal muscular atrophy (SMA).
- Cluster 2 (Intervention Technology): Encompasses assistive devices and smart environments, including terms such as assistive technology (MeSH), smart home systems, artificial intelligence (MeSH), ambient assisted living, robotic exoskeletons, eye tracking, and the Internet of Things (IoT).
- Cluster 3 (Clinical/Occupational Outcome): Target goals and frameworks, including occupational therapy (MeSH), rehabilitation, functional independence, and quality of life.

The exact syntax executed within each database query engine to intersect these clusters is presented in Table A1.

**Table A1: Database-Specific Search Strings and Structural Syntax**

| Database | Specific Search String |
| --- | --- |
| PubMed | ("assistive technology"[MeSH Terms] OR "smart home"[All Fields] OR "artificial intelligence"[MeSH Terms]) AND ("neuromuscular disorders"[MeSH Terms] OR "muscular dystrophy"[All Fields] OR "amyotrophic lateral sclerosis"[All Fields] OR "spinal muscular atrophy"[All Fields]) AND ("occupational therapy"[MeSH Terms] OR "rehabilitation"[All Fields]) |
| Scopus | TITLE-ABS-KEY(("assistive technology" OR "smart home" OR "ambient assisted living" OR "IoT") AND ("neuromuscular disorder*" OR "Duchenne" OR "ALS" OR "SMA") AND ("occupational therapy" OR "functional independence")) |
| Web of Science | TS=(("assistive technology" OR "robotic exoskeleton" OR "eye tracking") AND ("neuromuscular disease") AND ("occupational therapy" OR "quality of life")) |
| Google Scholar | ("assistive technology" OR "smart home systems") AND ("neuromuscular disorders") AND ("occupational therapy implications") custom range filter (2018–2026) |
